# Symmetry Dependence of the Continuum Coupling in the Chemi-ionization of Li(2^2^S_1/2_) by He(2^3^S_1_, 2^3^P_*J*_)

**DOI:** 10.1021/acs.jpca.3c00431

**Published:** 2023-05-15

**Authors:** Tobias Sixt, Taewon Chung, Frank Stienkemeier, Katrin Dulitz

**Affiliations:** † Institute of Physics, University of Freiburg, Hermann-Herder-Strasse 3, 79104 Freiburg, Germany; ‡ Institut für Ionenphysik und Angewandte Physik, Universität Innsbruck, 6020 Innsbruck, Austria

## Abstract

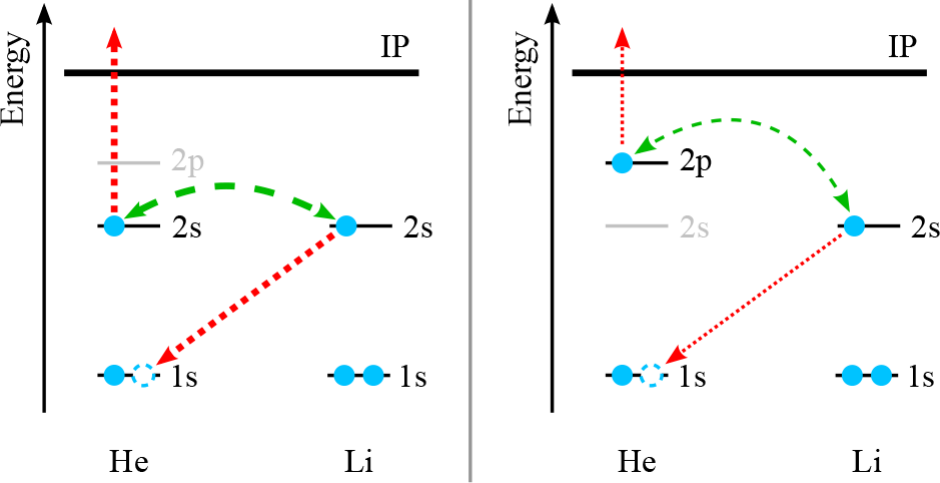

In the literature, the chemi-ionization of Li in the 2^2^S_1/2_ ground level by He in a metastable state is typically described as an electron transfer process in which an electron from the 2s orbital of Li is transferred to the 1s orbital of He while an electron from the 2s orbital of He is ejected. Therefore, one would not assume that the orbital of the valence electron of He strongly influences the coupling strength of the collision complex to the ionization continuum. However, we observe that the chemi-ionization rate is decreased when He is laser-excited from the metastable 2^3^S_1_ level to the 2^3^P_*J*_ level (with *J* = 0, 1, 2). A semiclassical treatment of the reaction dynamics reveals a strong dependence of the ionization rate on the reaction-channel-specific ionization width functions to which the observed decrease of the rate coefficients can be related to. The results are relevant for the improved understanding and control of chemi-ionization processes in merged beams and in traps.

## Introduction

Chemi-ionization occurs upon a collision of an excited, long-lived (“metastable”) atom A* with an atom B whose ionization energy is lower than the internal energy of A*:
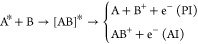
1In eq 1, the product pathways are referred to as Penning ionization (PI) and associative ionization (AI), respectively.

Chemi-ionization provides an ideal platform for exploring the quantum nature of chemical reactions.^1,2^ Previously, quantum effects in chemi-ionization processes have been demonstrated through the observation of barrier tunneling resonances and stereodynamic and quantum structural effects.^3−9^ The efficient suppression of chemi-ionization has become a key requirement for producing ultracold, dense samples of metastable rare gas atoms which are indispensable for the creation of ultracold molecules from these species and for chemistry studies at temperatures close to absolute zero. An efficient reduction of the chemi-ionization rate has been achieved for collisions of homonuclear rare-gas atoms in trapped samples by preparing the atoms in electron-spin-stretched states (see ref (2) and references therein). In these systems, chemi-ionization is strongly suppressed by electron-spin conservation due to the absence of outgoing channels with the same total spin quantum number as for the incoming channels. This effect has, for instance, enabled the Bose–Einstein condensation of ^4^He*^10,11^ as well as the production of a degenerate Fermi gas of ^3^He* and a Bose–Fermi mixture of ^4^He* and ^3^He*.^12^ However, for the heavier metastable rare gases, the anisotropic interaction induced by the spin–orbit coupling within the P levels has prevented a sufficiently strong suppression of the chemi-ionization process, and Bose–Einstein condensation has not yet been achieved.^13−15^

Two microscopic reaction mechanisms have been found to contribute to chemi-ionization: a short-range electron exchange process, often referred to as the direct mechanism, and a long-range virtual photon transfer process, often referred to as the indirect or radiative process (see, e.g., ref (1)). Electron exchange requires the transfer of an electron from B to the A* core which leads to the release of the excited electron of A*. The second mechanism involves the emission of a virtual photon by A* which ionizes B. Electron exchange is often considered to dominate the chemi-ionization process, particularly for systems in which the spin–orbit interaction is weak, such as the He*–Li system. Recently, charge exchange was also found to dominate the long-range ionization of Li atoms by excited He atoms at the surface of superfluid He nanodroplets.^16^

Anisotropic interactions within chemi-ionization complexes are responsible for a strong dependence of the chemi-ionization rate on the electron orbital arrangement. Experimentally, this dependency has been studied mostly in collisions involving Ne* by orienting the orbitals via laser excitation^17,18^ or via magnetic fields.^5,6,19,20^ A collision-energy-dependent competition between the two reaction mechanisms has been suggested as an explanation for the observed Ne*-Ar stereodynamics.^5,6^ Accurate theoretical descriptions of the state-to-state controlled chemi-ionization dynamics have been developed which take into account both electron exchange and virtual photon transfer.^8,21,22^ Additionally, anisotropic effects were found to dominate the chemi-ionization rates of H_2_ or HD by He(2^3^P_2_).^4^ Here, the interaction potential strongly depends on the orbital orientation of He(2^3^P_2_) and the orientation of the target molecular axis which results in either a strongly attractive or repulsive interaction potential at short internuclear distances between the collision partners. Since chemi-ionization at these short distances occurs with a probability close to unity, the reactivity is dominated by the long-range behavior of the interaction potential. In recent experiments, we have shown that the chemi-ionization process is strongly suppressed by orbital angular momentum conservation if the Li atoms are laser-excited to an anisotropic P level prior to the collision.^23^

This article is focused on the comparison of chemi-ionization rates for reactive collisions of Li in the 2^2^S_1/2_ ground level with He in the metastable 2^3^S_1_ level and with He in a fine-structure level within the 2^3^P manifold at thermal collision energies. We also apply a scheme for the all-optical control of the chemi-ionization rate, which has recently been applied to reactive collisions of Li(2^2^S_1/2_) with He(2^3^S_1_),^24^ in order to observe reactive collisions between spin-polarized Li(2^2^S_1/2_) and He(2^3^P_2_). This study substantially extends previous experimental and theoretical studies of the reactive He–Li system.^16,23−29^ Since the 2^3^P_2_ ← 2^3^S_1_ transition in He is also used for the laser cooling of the atom, the results of this study are of particular relevance for the efficient production of long-lived ultracold trapped He*–Li mixtures and subsequent production of ultracold HeLi molecules, for ultracold chemistry studies, and for precision measurements.

## Methods

### Experimental Methods

Most parts of the experimental setup have already been described elsewhere.^23,24,30−32^ In the following, we thus provide details about the most important parts of the setup and about those features which have specifically been implemented for this study.

**Figure 1:**
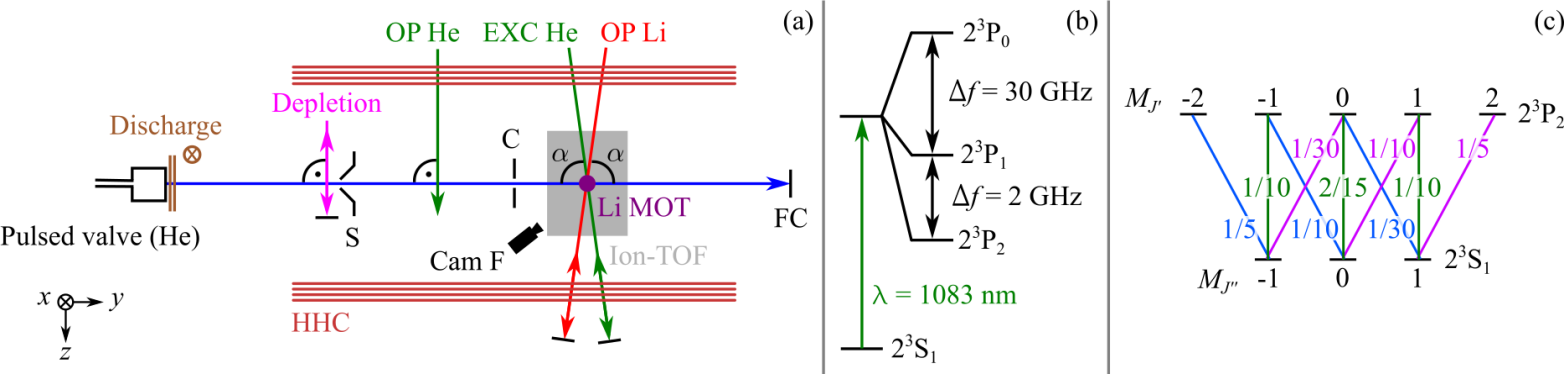
(a) Sketch of the experimental setup. A beam of metastable He is produced by a supersonic expansion through a pulsed valve. The velocity axis of the supersonic beam is indicated as a blue horizontal arrow. A homogeneous magnetic field in the *z* direction is generated by a pair of Helmholtz coils (HHC) located along the *xy* plane. The laser beams for the optical pumping of Li (OP Li) and for the 2^3^P_*J*_ ← 2^3^S_1_ excitation of He during the collisional interaction (EXC He) are tilted at an angle of α′ = 90° – α = 4° with respect to the *z* axis. Other abbreviations: skimmer (S), collimating aperture (C), CCD camera used to monitor the fluorescence of excited Li atoms (Cam F), ion-time-of-flight detector (Ion-TOF), Faraday-cup detector (FC), laser beam for the optical pumping of He(2^3^S_1_) (OP He), magneto-optical trap for ultracold Li atoms (Li MOT). (b) Schematic diagram of the relevant energy levels in triplet He which are coupled by laser radiation at λ = 1083, nm. (c) Possible magnetic sublevel transitions between the 2^3^S_1_ and 2^3^P_2_ levels of He. The laser polarizations required to drive the respective σ^+^, π and σ^–^ transitions are denoted by pink, green, and blue colors, respectively, and are labeled with their respective transition strengths. All subfigures are not to scale.

A sketch of the experimental setup is shown in Figure 1a. A pulsed atomic beam of He containing a fraction of ≈10^–4^ atoms in the metastable 2^3^S_1_ and 2^1^S_0_ levels^30^ is created by a supersonic expansion of ^4^He gas (10 bar backing pressure) through a room-temperature pulsed valve (30 μs pulse duration) into the vacuum and subsequent excitation of the atoms in an electron-seeded discharge. Flux and velocity of the beam of metastable He atoms (most probable forward velocity of *v* = 1820 m/s) are monitored by a gold-coated Faraday cup detector (FC). To efficiently deplete (by more than 99%) the population in the metastable 2^1^S_0_ level of He,^31^ the He beam is illuminated by laser light resonant with the 4^1^P_1_ ← 2^1^S_0_ transition wavelength near λ = 397 nm. Laser excitation is followed by a rapid spontaneous decay to the 1^1^S_0_ ground level of He which is not reactive. The He beam is directed into the reaction region which is predefined by the spatial extent of the ultracold (≈1 mK) cloud of ≈4 × 10^7^^7^Li atoms. The Li atoms are confined in a standard, three-dimensional magneto-optical trap (MOT). The MOT is fed by Li atoms which are emitted from a heated oven and translationally cooled inside a Zeeman slower. The Li atom number and the spatial dimensions of the Li cloud are monitored by imaging the fluorescence emitted by the trapped atoms onto a charge-coupled device (CCD) camera. Due to the negligibly small thermal velocity of the Li atoms, the collision energy of *E*_coll_ = 44 meV is determined by the forward velocity of the He supersonic beam. The Li^+^ and HeLi^+^ reaction products are detected simultaneously using an ion-time-of-flight (ion-TOF) detector which is built around the reaction region. Signals are acquired on a channel-electron-multiplier (CEM) in counting mode.

In order to examine the change in the chemi-ionization rate upon excitation of He from the 2^3^S_1_ level to a 2^3^P_*J*_ level (with *J* = 0, 1, 2), the reaction region is illuminated by a laser beam resonant with the respective 2^3^P_*J*_ ← 2^3^S_1_ transition wavelength near λ = 1083 nm (cf. Figure 1b). This laser excitation process is referred to as “EXC” hereafter. The laser beam is collimated to a Gaussian waist diameter of 6 mm which is much larger than the ≈1 mm diameter of the Li cloud in the MOT and ensures a nearly uniform laser intensity distribution within the reaction region. For technical reasons, the angle between the laser axis and the quantization axis (see below) is α′ = 4°. Before entering the vacuum chamber, the laser polarization is adjusted using a quarter-wave plate. Since the windows are antireflection-coated for a wavelength of 671 nm, approximately 10% of the laser power at λ = 1083 nm is reflected at each window. These reflection losses are accounted for in the further analysis. Additionally, the laser beam is retro-reflected at the outside of the vacuum chamber to further increase the laser intensity in the interaction volume. All measurements are taken in a toggled manner, where the laser is blocked by a fast mechanical shutter at every second valve opening event. Furthermore, ion signal contributions arising from He(2^3^S_1_, 2^3^P_*J*_)-self-collisions and He(2^3^S_1_, 2^3^P_*J*_)-background-gas collisions, which occur both in the absence and in the presence of the Li cloud, are recorded in separate measurements and used for background subtraction.

In experiments with EXC to the 2^3^P_2_ level of He, optical pumping (OP) is used to prepare the relative spin orientations of the reaction partners beforehand. For this, the apparatus is surrounded by a pair of Helmholtz coils which produces a homogeneous magnetic bias field of *B*_*z*_ ≈ 3*G*. This magnetic bias field defines the quantization axis for the He atoms in the 2^3^S_1_ and 2^3^P_2_ levels and for the ground-state Li atoms during OP as well as during the reaction process. Moreover, the magnetic fields and lasers for the MOT and for the Zeeman slower are switched off 1 ms before the pulsed valve is triggered so that the homogeneity of the magnetic bias field is not disturbed during OP. The spin orientation of He in the 2^3^S_1_ level is prepared by illuminating the atoms with circularly polarized laser light resonant with the 2^3^P_2_ ← 2^3^S_1_ transition wavelength near λ = 1083 nm.^32^ A schematic drawing showing the magnetic sublevel structure of both levels as well as the possible transitions in between the levels is presented in Figure 1c. After OP, more than 90% of the He atoms populate the *M*_*J*_ = +1 or *M*_*J*_ = −1 sublevel depending on whether the circularly polarized laser light is right-handed or left-handed, respectively. On the other hand, the spin orientation of the Li atoms is manipulated by illuminating the atoms with circularly polarized laser light which consists of two nearby wavelength components resonant with the 2^2^*P*_1/2_, *F* = 2 ← 2^2^S_1/2_, *F* = 2 and 2^2^*P*_1/2_, *F* = 2 ← 2^2^S_1/2_, *F* = 1 transition wavelengths near λ = 671 nm. By decoupling the electron spin from the nuclear spin contribution within the 2^2^S_1/2_, *F* = 2 level, the population of Li atoms in the designated *M*_*S*_ = +^1^/_2_ (*M*_*S*_ = −^1^/_2_) spin-sublevel is determined as >90% after OP with right-handed (left-handed) circularly polarized laser light.^24^

### Theoretical Methods

Since the sub-ps collision time^41^ is much faster than the natural lifetime of He-(2^3^P) (τ ≈ 98 ns^33^), the EXC process can be considered to occur prior to and independent from the collision process. Thus, the He*–Li chemi-ionization kinetics are modeled using
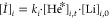
2where *k*_*i*_ denotes the rate coefficient for a specific electronic-state combination *i*, [*I*]_*i*_ is the state-dependent density of the product ions (i.e., the sum of Li^+^ and HeLi^+^), and  is the state- and time-dependent density of He* (here, both He(2^3^S_1_) and He(2^3^P_*J*_) are referred to as He*) while the density of ground-state Li, [Li]_*i*,0_, is assumed to be constant. Due to the fact that the absolute densities of the two species are difficult to determine in experiments, we only provide chemi-ionization rate ratios for He(2^3^P_*J*_)–Li(2^2^S_1/2_) vs He(2^3^S_1/2_)–Li(2^2^S_1/2_) collisions which are obtained from the respective time-integrated ion yield ratios in the reaction region after background subtraction. In addition to that, the fractional 2^3^P_*J*_ level population produced by EXC is taken into account. This yields
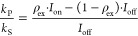
3where *k*_P_ and *k*_S_ denote the chemi-ionization rate coefficients for He(2^3^P_*J*_)–Li(2^2^S_1/2_) and He(2^3^S_1/2_)–Li(2^2^S_1/2_) collisions, respectively. Further, ρ_ex_ is the fractional population of He in the corresponding 2^3^P_*J*_ level and *I*_on_ (*I*_off_) is the ion yield in the presence (absence) of EXC.

Microscopically, the chemi-ionization rate coefficients can be quantified by describing the nuclear motion within a complex potential for each reactive channel. A detailed theoretical description is given in ref (34). To calculate total ionization rates at thermal collision energies, it is sufficient to describe the nuclear motion of the collision complex in a semiclassical way. The complex potential is described within the center-of-mass frame of the collision complex and can be expressed as
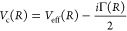
4where *R* is the internuclear distance. The first term *V*_eff_(*R*) = *V*_0_(*R*) + *V*_*l*_(*R*) determines the nuclear motion within of the collision complex. It consists of a molecular term *V*_0_(*R*) that results from the coupling of the atomic orbitals and a centrifugal part *V*_*l*_(*R*) = *ℏ*^2^*l*(*l* + 1)/(2*μR*^2^) which accounts for the rotational motion of the nuclei. Here, *l* is the rotational angular momentum quantum number and μ is the reduced mass. In the second term of eq 4, Γ(*R*) describes the coupling strength of the collision complex to the ionization continuum. In this case, the total probability for a transition to the continuum of states can be determined by integrating all partial probabilities for all infinitesimal small internuclear distance sections while the two nuclei move from infinite separation to the distance of closest approach *R*_0_ and back,
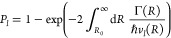
5The relative velocity *v*_*l*_(*R*) is determined by solving the equations of motion for *V*_eff_(*R*) including the boundary condition .

The total cross section is calculated as follows
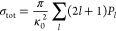
6with κ_0_^2^ = 2*μE*_coll_/ℏ. The chemi-ionization rate coefficient *k*_*i*_ is directly related to σ_tot_ via *k*_*i*_ = σ_tot_·*v*_*l*_(*R* → *∞*). The series in *l* in eq 6 is truncated when *P*_*l*_ < 10^–8^.

Since only the asymptotic state combinations of the collision partners can be prepared in the experiment, a precise knowledge of the contributions of the specific reactive channels to the prepared asymptotic states is necessary in order to compare the experimental results with the results from semiclassical trajectory calculations. The channels connected to the He(2^3^S_1_)–Li(2^2^S_1/2_) asymptote are of ^2^Σ and ^4^Σ symmetry, and the channels linked to the He(2^3^P_*J*_)–Li(2^2^S_1/2_) asymptote are of ^2^Σ, ^4^Σ, ^2^Π, and ^4^Π symmetry. We account for the principles of electron-spin conservation which have recently been shown to hold for the He*–Li system.^24^ Specifically, we assume that the channels of doublet symmetry react while the channels of quartet symmetry do not, since the reaction products are exclusively formed in states of ^2^Σ symmetry.

In our semiclassical trajectory calculations, we use the respective potential energy curves *V*_0_(*R*) for the doublet channels of the He(2^3^S)–Li(2^2^S_1/2_) and He(2^3^P)–Li(2^2^S_1/2_) systems, and Γ(*R*) for the He(2^3^S_1_)–Li(2^2^S_1/2_) ^2^Σ channel which were previously calculated by Movre et al.^28^ However, the potential-energy-curve calculations by Movre et al.^28^ did not resolve the fine-structure slitting for the He(2^3^P_*J*_)–Li(2^2^S_1/2_) asymptotic states (i.e., only a single asymptote is given for each reactive channel of different symmetry). Therefore, we do not treat the individual *J* levels of the 2^3^P manifold of He in our calculations. In this case, accounting for the electron-spin statistics, ^1^/_3_ of the channels connected to each asymptote can be considered reactive. Consequently, spin-statistical effects cancel out when only relative rates *k*_P_/*k*_S_ for He(2^3^P)–Li(2^2^S_1/2_) chemi-ionization vs He(2^3^S_1_)–Li(2^2^S_1/2_) chemi-ionization are considered. Moreover, the collision complex prepared at the He(2^3^P)–Li(2^2^S_1/2_) asymptote has the possibility to reactively scatter via a ^2^Σ and a ^2^Π potential. Since the ^2^Σ channel is doubly degenerate, and the ^2^Π channel is 4-fold degenerate, we take into account that the probability for reactive scattering via the ^2^Π channel is twice as high than for the ^2^Σ channel.

To obtain a continuously differentiable representation of *V*_0_(*R*), we fit Morse-long-range (MLR) functions^35^ to these potential energy curves. The MLR function has already been successfully used to represent the ^4^Σ potential energy curves of different He(2^3^S_1_)–alkali atom systems and is expressed as^36^
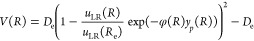
7with
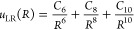
8
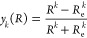
9and
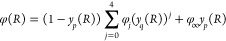
10For each reactive channel, we use the long-range coefficients *C*_6_, *C*_8_ and *C*_10_ given in ref (37), and we use the same parameters *p* = *q* = 4 as used for the He(2^3^S_1_)–Li(2^2^S_1/2_) ^4^Σ channel previously.^36^ Therefore, the fitting parameters are *D*_e_, *R*_e_ and ϕ_*j*_ with (*j* = 0, 1, ..., 4), while ϕ_*∞*_ = ln(2*D*_e_/*u*_LR_(*R*_e_)). All parameters for the fitted potentials can be found in the Supporting Information (see Table S1). The agreement between the fitted MLR functions and the potential data is better than 50 meV for the reactive He(2^3^S_1_)–Li(2^2^S_1/2_), ^2^Σ channel and better than 20 meV for the other channels.

In the special case in which a He atom in the 2^3^P_2_ level is prepared in a spin-stretched quantum state  with respect to the quantization axis *z*, the contributions from orbital angular momentum and electron spin can be separated to . However, the symmetry of the reactive channels is defined with respect to the collision axis which is given by the direction of the forward velocity vector of the He beam (*x* axis). Angular momentum states within the two reference frames are connected via the following transformation
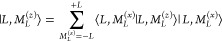
11
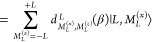
12where the overlap coefficients can be identified as the Wigner D-matrix elements for a rotation of the coordinate system by β = 90°.^38^ Considering that Li(2^2^S_1/2_) has zero orbital angular momentum (*L* = 0), the orbital angular momentum quantum numbers of the quasi-molecule are directly related to the quantum states of He(2^3^P_2_) by . Therefore, in this case, the contributions of the respective reaction channels can be found using
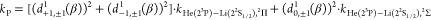
13
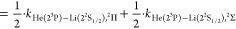
14Note that  because only one reactive channel is connected to the He(2^3^S_1_)–Li(2^2^S_1/2_) asymptote.

## Experimental Results

### Unpolarized Atoms

**Figure 2:**
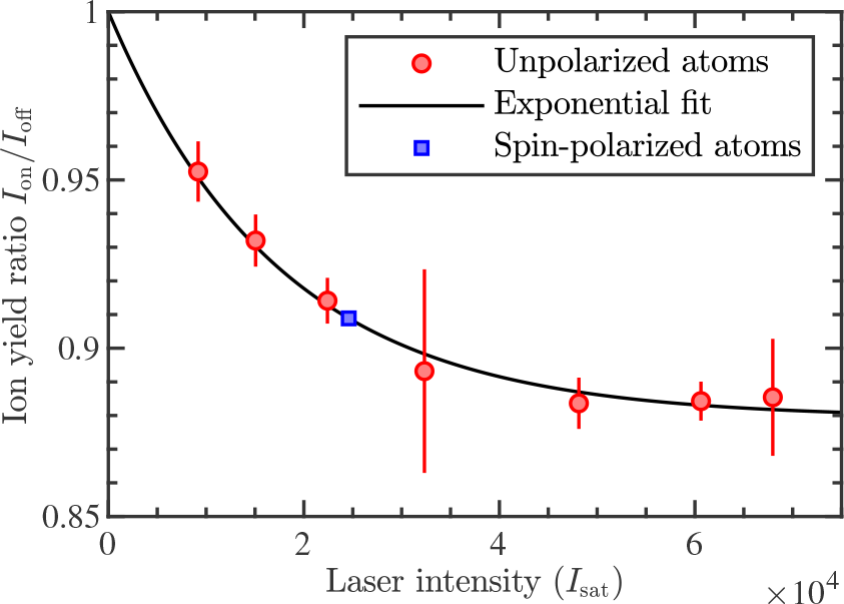
Ion yield ratios for He*–Li chemi-ionization as a function of laser intensity for EXC. Here, the laser resonantly excites the 2^3^P_2_ ← 2^3^S_1_ transition in He. The laser intensity is expressed in units of the resonant saturation intensity *I*_sat_. Data points obtained using unpolarized (spin-polarized) atoms are labeled with red circles (blue squares). An exponential fit to the data which is used to extract the population of He in the 2^3^P_2_ level is shown as a solid black line. The error bars are statistical only.

To determine the relative rates *k*_P_/*k*_S_ for unpolarized collision partners, the lasers for the OP of He and Li are blocked and the magnetic fields of the MOT and of the Zeeman slower remain turned on during the collision process so that there is no uniform quantization axis. Hence, all measured values can be viewed as independent from the laser polarization direction, and we expect an equal population distribution across all involved sublevels of both collision partners. Figure 2 shows ion yield ratios for He*–Li chemi-ionization as a function of laser intensity for EXC. Here, a laser beam resonant with the 2^3^P_2_ ← 2^3^S_1_ transition in He is used for electronic excitation. Due to the tilt of the laser beam with respect to the *z* axis, a Doppler shift of ≈100 MHz is induced by the forward velocity of the He supersonic beam. Thus, the laser intensity required to saturate the transition is much larger than the saturation intensity *I*_sat_ = 0.16 mW/cm^2^ which is determined from the natural lifetime of the 2^3^P levels in He.^33^ However, at sufficiently high laser intensity, the ion yield ratio saturates. This implies that the population of He in the 2^3^P_2_ level reaches ρ_ex_ = 50%. In this regime, it is possible to quantify the experimental results according to eq 3 for all fine-structure levels 2^3^P_0,1,2_ of He. The results are summarized in the top part of Table 1, which suggests that the chemi-ionization rate ratio *k*_P_/*k*_S_ is consistently below 1 for all the fine-structure levels of the 2^3^P manifold of He (i.e., *k*_P_ < *k*_S_ throughout).

**Table 1:** Experimentally Obtained He*–Li Chemi-ionization Rate Ratios *k*_P_/*k*_S_ for Different Fine-Structure Levels of the 2^3^P Manifold of He and Different Laser Polarizations Used for OP and EXC at a Collision Energy of *E*_coll_ = 44 meV^a^

	Laser polarization			
He term after EXC	OP Li (*M*_*S*_)	OP He (*M*_*S*_)	EXC He (*M*_*J*_)	*k*_P_/*k*_S_
2^3^P_0_	–	–	linear	0.89_–2_^+1^
2^3^P_1_	–	–	linear	0.82_–3_^+2^
2^3^P_2_	–	–	linear	0.79_–3_^+2^
2^3^P_2_	σ^–^ (−1/2)	σ^+^ (+1)	σ^+^ (+2)	0.74_–5_^+5^
2^3^P_2_	σ^+^ (+1/2)	σ^–^ (−1)	σ^–^ (−2)	0.74_–6_^+6^
2^3^P_2_	σ^+^ (+1/2)	σ^+^ (+1)	σ^+^ (+2)	0.95_–11_^+11^
2^3^P_2_	σ^–^ (−1/2)	σ^–^ (−1)	σ^–^ (−2)	0.67_–10_^+10^

a The values in parentheses give the magnetic projection quantum numbers of the spin *M*_*S*_ or of the total angular momentum *M*_*J*_ which are populated for each polarization of the OP lasers or of the EXC laser, respectively. The given error bounds are determined from the statistical fluctuation of the ion yields and the estimated 5% uncertainty of the population in the corresponding He(2^3^P_*J*_) level, ρ_ex_.

### Spin-Polarized Atoms

Chemi-ionization rate ratios *k*_P_/*k*_S_ are also determined for spin-polarized atoms (see Experimental Methods). Using the 2^3^P_2_ ← 2^3^S_1_ transition wavelength in He and σ^+^ (σ^–^) polarized light for EXC, a fraction of the He atoms is prepared in the 2^3^P_2_, *M*_*J*_ = +2 (*M*_*J*_ = −2) sublevel inside the reaction region. Yet, the limiting power of our laser system and laser power splitting to provide light for both OP and EXC meant that experiments could not be done in a regime where the population in the 2^3^P_2_ level of He is saturated. Thus, the intensity-dependent ion yield ratio in Figure 2 is fit to an exponential function, and we use an interpolated value of ρ_ex_ = 37(5)% for the experiments with spin-polarized atoms.

**Figure 3:**
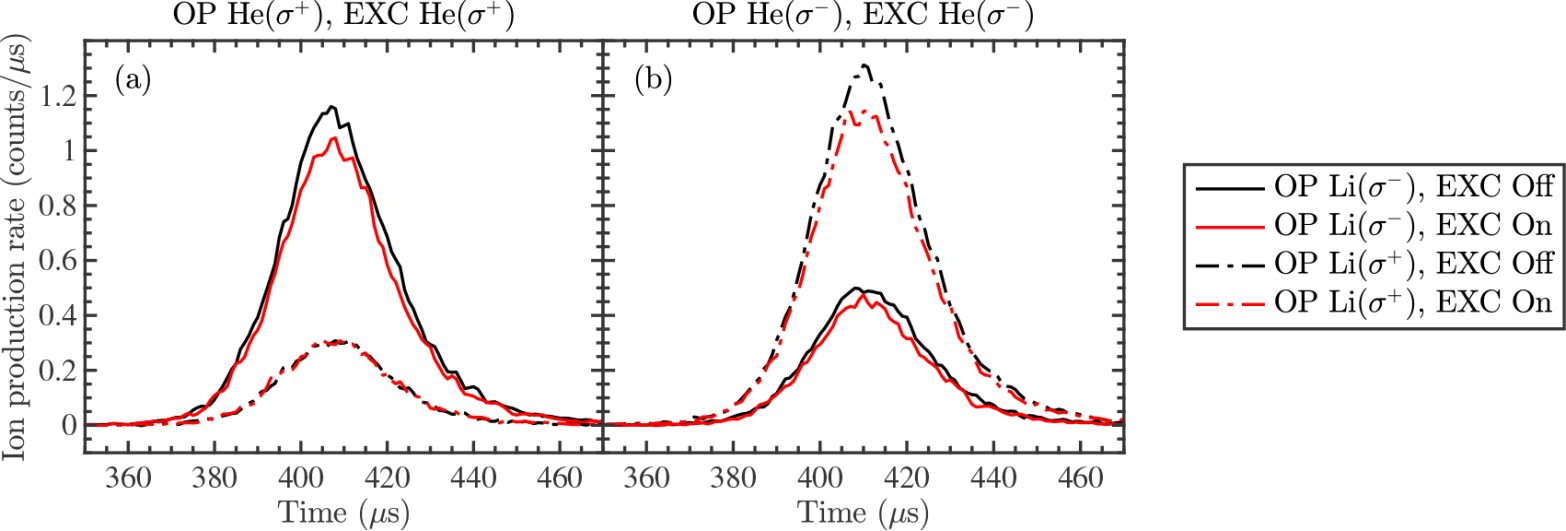
Measured ion count rates as a function of He time-of-flight to the reaction region for He*–Li chemi-ionization with spin-polarized atoms in the presence or absence of EXC indicated by red or black colors, respectively. The laser polarizations for the OP and EXC of He are set to (a) σ^+^ and (b) σ^–^. The laser polarization for the OP of Li is set to σ^–^ or σ^+^ for the solid or dash-dotted lines, respectively.

The measured ion production rates for spin-aligned and spin-antialigned atoms are shown in Figure 3. The rate coefficient ratios obtained using eq 3 are given in Table 1. As shown in the figure and in the table, the chemi-ionization rate ratio for spin-antialigned atoms is—within the error margins—the same as for unpolarized atoms. For spin-aligned atoms, the rate ratio is mostly determined by the remaining unpolarized atoms within the reaction volume and is thus different compared to spin-antialigned atoms. The fact that none of the ratios exceeds 1 implies that electron spin conservation, which was previously observed for the He(2^3^S_1_)–Li(2^2^S_1/2_) collision system,^24^ also holds for the He(2^3^P_2_)–Li(2^2^S_1/2_) collision system and that anisotropic effects (e.g., induced by spin–orbit coupling) are negligible.

## Discussion

To obtain further insight into the underlying reaction mechanism, we compare the experimental values with the results from semiclassical trajectory calculations. The latter are summarized in Table 2. We restrict the comparison to the experimental results obtained using spin polarized atoms, because the assignments of the different reactive channels within the He(2^3^P)–Li(2^2^S_1/2_) asymptote to the different *J* states are not clear for the unpolarized atoms (see Table 1). In addition, only the rate coefficient ratios measured for opposite handedness of circular polarization of OP He and OP Li are of importance here, because only this reaction is not forbidden by spin conservation.

**Table 2:** Rate Coefficients, Classical Turning Points *R*_0_ and Highest Partial Wave Quantum Numbers *l*_max_ for the He*–Li System at a Collision Energy of *E*_coll_ = 44 meV Obtained Using Semiclassical Trajectory Calculations^a^

Reactive channel *i*	*R*_0_ (*a*_0_)	*l*_max_	*k*_*i*_ (10^–9^cm^3^/s)	*k_i_*/*k*_He(2^3^S_1_)–Li(2^2^S_1/2_),^2^Σ_
He(2^1^S_0_)–Li(2^2^S_1/2_), ^2^Σ	4.9	63	2.33	
He(2^3^S_1_)–Li(2^2^S_1/2_), ^2^Σ	3.8	61	2.60	
He(2^3^P)–Li(2^2^S_1/2_), ^2^Σ	4.8	72	2.69	
He(2^3^P)–Li(2^2^S_1/2_), ^2^Π	3.8	57	2.49 [1.16]	
He(2^3^P)–Li(2^2^S_1/2_), polarized atoms			2.59 [1.93]	1.00 [0.74]

a The reactive channels in the first part of the table are labeled with their asymptotic atomic and quasi-molecular terms. The second part of the table gives the rate coefficients and rate coefficient ratios of the experimentally prepared mixture of reactive channels as determined using eq 14. The values in square brackets are calculated using Γ(*R*)/3 for the He(2^3^P)–Li(2^2^S_1/2_), ^2^Π channel.

**Figure 4:**
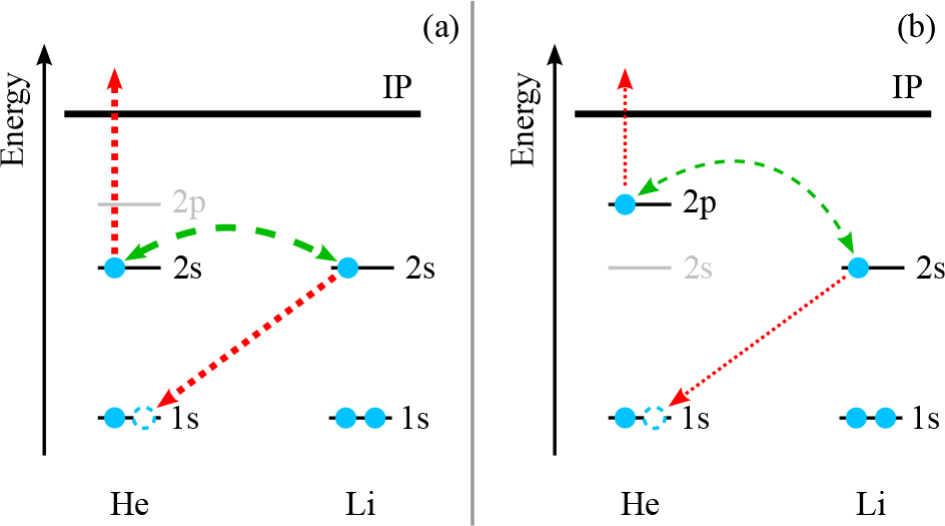
Schematic drawings of the electron exchange process leading to chemi-ionization in (a) He(2^3^S)–Li(2^2^S_1/2_) and (b) He(2^3^P)–Li(2^2^S_1/2_) collisions. In each subfigure, the electron transfer within the collision complex is represented by dotted red arrows, and the coupling between the involved electrons is denoted by a dashed green double arrow. Differences in the relative coupling strengths are indicated by the corresponding arrow thicknesses.

As a first approximation, we calculated the rate coefficients using the known ionization width Γ(*R*) for the He(2^3^S)–Li(2^2^S_1/2_), ^2^Σ system^28^ for all reactive channels. This may be justified because the ionization width follows an exponential scaling as a function of *R*,^28^ which is indicative of a short-range electron exchange-process between the valence electron of the Li atom and the He^+^ core hole. He*–Li chemi-ionization may thus be described as a charge-transfer-like process from He^+^–Li to He–Li^+^ (see Figure 4a), which is independent of the valence electron of He*.

This assumption is further supported by experimental and theoretical results for the chemi-ionization rate ratio for He(2^3^S_1_)–Li(2^2^S_1/2_) vs He(2^1^S_0_)–Li(2^2^S_1/2_) collisions given by
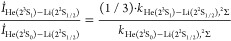
15
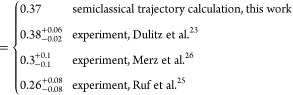
16The factor ^1^/_3_ in eq 16 is due to the fact that a He(2^3^S_1_)–Li(2^2^S_1/2_) collision complex can be formed within the doubly degenerate reactive ^2^Σ and the 4-fold degenerate nonreactive ^4^Σ channels.

If the same functional form of Γ(*R*) is used for all the channels, then the semiclassical trajectory calculation yields a rate coefficient ratio *k*_P_/*k*_S_ = 1.00 for the spin-polarized atoms (see Table 2) on account of *R*_0_ and *l*_max_ being nearly identical for the He(2^3^S_1_)–Li(2^2^S_1/2_), ^2^Σ and the He(2^3^P)–Li(2^2^S_1/2_), ^2^Π channels. Here, *l*_max_ is the maximum partial wave quantum number for which the collision energy exceeds the centrifugal barrier. In comparison, the He(2^3^P)–Li(2^2^S_1/2_), ^2^Σ channel exhibits a 26% larger *R*_0_ and an ≈18% larger *l*_max_. Since a larger *R*_0_ decreases the channel-specific chemi-ionization rate coefficient while a larger *l*_max_ increases it, the effects of both quantities cancel each other out thus resulting in similar rate coefficients for all reactive channels. This is different from the strong anisotropy reported for the potential energy functions of the He(2^3^P)–H_2_ system.^4^ However, the calculated rate coefficient ratios are not compatible with the experimental value *k*_P_/*k*_S_ = 0.74 in Table 1. The calculated ratio *k*_P_/*k*_S_ can be brought into closer agreement with the experimental results by linearly scaling the ionization widths  and  for the reactive He(2^3^P)–Li(2^2^S_1/2_), ^2^Σ and ^2^Π channels, respectively, by a constant factor so that
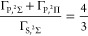
17where  is the known ionization width for the reactive He(2^3^S_1_)–Li(2^2^S_1/2_), ^2^Σ channel. If we assume that  and use a ratio of  (from eq 17), we obtain the calculated ratios *k*_P_/*k*_S_ = 0.74 for reactive collisions of polarized atoms (see values in brackets in Table 2) which are close to the experimental values. Accurate theoretical calculations of the ionization widths are highly desired to provide more quantitative insights.

A decrease of the chemi-ionization rate upon the laser excitation from an S to a P level has already been reported by us for He(2^3^S_1_)–Li(2^2^S_1/2_) vs He(2^3^S_1_)–Li(2^2^P_1/2,3/2_) collisions.^23^ We have interpreted these previous results in terms of a suppression of chemi-ionization due to the conservation of the orbital angular momentum projection onto the internuclear axis, Λ.^23^ The previous observations are consistent with the picture of an electron-exchange process which relies on the constructive overlap of atomic orbitals. In that case, the decrease of the chemi-ionization rate upon excitation of Li from an S to a P level can be explained by the vanishing overlap between the 1s core hole orbital of He and the 2p_*x*, *y*_ orbitals of Li. In the present case, the excited electron of He does not contribute to the electron exchange process. However, an energetic coupling (e.g., by a virtual photon) between the exchanging electron of Li and the excited electron of He is necessary for the latter to leave the collision complex (see Figure 4).

When the valence electron in He is located in a 2p orbital, this coupling might be weakened by anisotropic effects. This suggests that, in the present case, the quantum states formed within the reactive He(2^3^P)–Li(2^2^S_1/2_), ^2^Π channel are more weakly coupled to the quantum states of the ionized complex (which are exclusively of ^2^Σ symmetry) than the quantum states formed within the reactive He(2^3^P)–Li(2^2^S_1/2_), ^2^Σ channel. This justifies the use of different ionization widths for the ^2^Σ and ^2^Π symmetries of the He(2^3^P)–Li(2^2^S_1/2_) channel.

The difference in ionization widths is in line with prior work on a related process, the interatomic Coulombic decay (ICD) of weakly bound systems, where energy transfer mediated by a virtual photon often dominates over charge exchange. For a Ca^+^He cluster, it was found that the ICD width (for virtual photon transfer) for a state of ^2^Σ symmetry was up to four times larger than the width for a state of ^2^Π symmetry.^39,40^ This was interpreted as a preferred ionization of the He atoms along the direction of a particular Ca 3p orbital. A similar behavior has recently been predicted for the ICD widths of the He(2^2^P_1_)–Li(2^2^S_1/2_) system at very large internuclear distances, where .^29^ The ratio can also be explained by the ratio of Clebsch–Gordan coefficients (i.e., ^4^/_3_ for the ^2^Σ and ^1^/_3_ for the ^2^Π states^40^).

An experimental verification of the described mechanism might be possible by rotating the quantization axis against the collision axis. If both axes are parallel to each other, then β = 0 in eq 13; the collision would take place purely via the ^2^Π channel. However, an experimental realization of this idea is not feasible in our setup, as this would require the lasers for the OP and EXC of He to point along the forward velocity axis of the He supersonic beam. This arrangement would lead to a Δν ≈ 2 GHz Doppler shift of the resonance frequency and a Doppler broadening of *δν* ≈ 150 MHz which would have to be compensated for (e.g., by a laser frequency chirp).

## Conclusions

We have observed chemi-ionizing collisions between He in the laser-excited 2^3^P_*J*_ levels (*J* = 0, 1, 2) and Li in the 2^2^S_1/2_ level and found a decrease of the ionization rate for He(2^3^P_*J*_)–Li(2^2^S_1/2_) collisions compared to He(2^3^S_1_)–Li(2^2^S_1/2_) collisions. The results from a semiclassical treatment of the collision dynamics can be brought into agreement with the experimental results by assuming that the coupling strength to the ionic continuum states is smaller for the He(2^3^P_*J*_)–Li(2^2^S_1/2_) states of ^2^Π symmetry than for those of ^2^Σ symmetry. In addition, we have implemented spin-state control for He(2^3^P_2_)–Li(2^2^S_1/2_) collisions and found that the level of spin suppression for this system is the same as for the He(2^3^S_1_)–Li(2^2^S_1/2_) system. The results imply that the laser cooling and co-trapping of He* and Li in a two-component MOT is feasible: the optical cycling of He via the 2^3^P_2_ ← 2^3^S_1_ transition will not induce more losses from chemi-ionization.

## Supporting Information Available

MLR fit parameters (Table S1) showing the best fit parameters for representing the potential energy curves for the He*–Li collision system by the MLR function in eq 7, where the reactive channels are labeled by the asymptotic terms of the collision partners and the corresponding quasi-molecular terms and the values for *C*_6_, *C*_8_, and *C*_10_ are taken from ref. .^36^ (PDF)
