# Supplementary material for: Symmetry Dependence of the Continuum Coupling in the Chemi-ionization of Li(22S1/2) by He(23S1, 23PJ)
Source: J Phys Chem A. 2023 May 15;127(20):4407–14. doi: 10.1021/acs.jpca.3c00431 (PMC10226102; doi:10.1021/acs.jpca.3c00431)
Supplement: Supplementary file 1 — jp3c00431_si_001.pdf [file jp3c00431_si_001.pdf]

Symmetry Dependence of the Continuum Coupling in the Chemi-ionization of Li(2^2S$_{1/2}$) by He(2^3S$_1$, 2^3P$_J$)

Tobias Sixt,[†] Taewon Chung,[†] Frank Stienkemeier,[†] and Katrin Dulitz[*,†,‡]

†*Institute of Physics, University of Freiburg, Hermann-Herder-Str. 3, 79104 Freiburg, Germany*

‡*Institut für Ionenphysik und Angewandte Physik, Universität Innsbruck, 6020 Innsbruck, Austria*

E-mail: katrin.erath-dulitz@uibk.ac.at

Supporting Information Available

MLR Fit Parameters

Table S1: Best fit parameters for representing the potential energy curves for the He*-Li collision system by the MLR function in Eq. (7). The reactive channels are labeled by the asymptotic terms of the collision partners and the corresponding quasi-molecular terms. The values for C_6, C_8 and C_{10} are taken from Ref.[1]

Parameter	He(2^3S_1)-Li($2^2S_{1/2}$), $^2\Sigma$	He(2^1S_0)-Li($2^2S_{1/2}$), $^2\Sigma$	He(2^3P)-Li($2^2S_{1/2}$), $^2\Sigma$	He(2^3P)-Li($2^2S_{1/2}$), $^2\Pi$
R_e [a.u.]	5.55609247	7.24770048	6.73540368	5.56280892
D_e [a.u.]	0.03140818	0.01118099	0.01955991	0.02357172
ϕ_0	−3.31021363	−3.35584089	−3.19880967	−3.18894845
ϕ_1	0.46936106	0.23647785	0.03711605	−0.26659077
ϕ_2	−0.11910569	1.85370133	−1.12894795	0.98349326
ϕ_3	−2.4712118	0.96471192	−1.17018335	2.39491642
ϕ_4	−3.79405849	−1.8736792	−1.69881596	−0.23483651
C_6 [a.u.]	2090	3504	7153	2609
C_8 [a.u.]	132600	263300	561700	67900
C_{10} [a.u.]	12800000	30180000	66200000	4464000

References

(1) Zhang, J.-Y.; Tang, L.-Y.; Shi, T.-Y.; Yan, Z.-C.; Schwingenschlögl, U. Long-range interactions between excited helium and alkali-metal atoms. *Phys. Rev. A* **2012**, *86*, 064701.
